# Vaso-Motors of the Lymphatics

**Published:** 1882-03

**Authors:** 


### Vaso-Motors of the Lymphatics.

MM. Paul Bert and Laffont have discovered the vaso-motors of the chyliferous glands. They opened the abdomen of an animal in warm water, while the process of digestion was in full play. The lacteal then reveal themselves in the form of white cords, and it suffices to simply excite the solar plexus or the great splanchnic nerve, to render visible the nodosities that form along these vessels. These experiments were announced to the Societe de Biologie, April 2, and repeated in *Le Progress Medical*, No. 15.

